# Hyperhomocysteinemia and Ischemic Stroke: A Potential Dose-Response Association—A Systematic Review and Meta-analysis

**DOI:** 10.1055/s-0041-1735978

**Published:** 2021-09-24

**Authors:** Marte Holmen, Anne-Mette Hvas, Johan F. H. Arendt

**Affiliations:** 1 Department of Clinical Biochemistry, Aarhus University Hospital, Aarhus, Denmark; 2 Department of Clinical Medicine, Aarhus University, Aarhus, Denmark

**Keywords:** stroke/prevention, cerebrovascular disease, homocysteine, meta-analysis

## Abstract

**Background and Purpose** Previous studies suggest an association between increased homocysteine (Hcy) and risk of ischemic stroke. Yet, it remains unknown whether a dose-response association exists between Hcy levels and risk of ischemic stroke.

**Methods** Systematic literature searches were performed in PubMed, Embase, Scopus, and Web of Science. Inclusion criteria were studies investigating ischemic stroke risk in an adult population with measured Hcy levels. We computed odds ratios (ORs) for a 5 µmol/L increase in Hcy levels using a random effects meta-analysis.

**Results** In total, 108 studies met the inclusion criteria of which 22 were rated as high-quality studies, and 20 studies included a dose-response analysis. Hcy levels were analyzed either as a continuous or categorical variable. The majority of the studies found an increased risk of ischemic stroke when comparing the highest-to-lowest Hcy strata. A graded association was observed over the Hcy strata, indicating a dose-response association, with the most apparent effect when Hcy levels exceeded approximately 15 µmol/L. No studies explored a potential nonlinear association between Hcy levels and ischemic stroke. Six studies were included in a meta-analysis, showing an OR of 1.43 (95% confidence interval [CI]: 1.28–1.61) per 5 µmol/L increase in Hcy levels.

**Conclusion** This review and meta-analysis indicate a dose-response association between Hcy levels and ischemic stroke. An evident increase in effect measures was observed when Hcy levels exceeded 15 µmol/L, indicating a nonlinear association between ischemic stroke and Hcy levels. This nonlinear association warrants further study.

This study is registered with clinical trial ( *https://www.crd.york.ac.uk/prospero/* ; unique identifier: CRD42019130371).

## Introduction

The comprehension that elevated homocysteine (Hcy) in plasma might predispose to arterial or venous thromboembolism emerged more than 40 years ago, when patients with homocysteinuria were observed to have a high risk of early vascular disease. 1 This led to extensive research regarding the role of Hcy in cardiovascular disease (CVD) and whether elevated Hcy is a modifiable risk factor.

Elevation of Hcy levels may be caused by several factors, including deficiency of vitamin B6, folate, and/or vitamin B12, due to insufficient intake or absorption, renal insufficiency, several drugs, 2 lifestyle factors, such as smoking and alcohol intake, or genetic factors. 3

Among fasting individuals, normal Hcy levels commonly range from 5 to 15 µmol/L. 4 Animal studies have demonstrated that elevated Hcy levels leads to complex changes within the blood vessel wall, with increased oxidative stress, proinflammatory effects, and endothelial dysfunction, indicating that an association between increased Hcy and CVD is biologically plausible. 5 6 7 Several studies have investigated the potential association between elevated Hcy concentration and risk of CVD, including stroke, but results are inconsistent. 8 9 10

The Norwegian Vitamin Trial indicated that treatment with folic acid and vitamin B combination therapy effectively lowered Hcy levels by 28%, but no effect was found on the incidence of ischemic stroke. 11 The Vitamin Intervention for Stroke Prevention trail demonstrated similar results, with no significant reduction in the risk of stroke among patients treated with B-vitamin combinations. 12 In contrast, the China Stroke Primary Prevention Trial reported a 24% risk reduction for ischemic stroke in the group that received folic acid treatment. 13 While, a Cochrane review from 2017 found a small reduction in risk of stroke in patients treated with B12, folate and B6 vitamins compared with patients receiving placebo. 14

Systematic reviews of observational studies have reported a dose-response related association between Hcy levels and the risk of stroke, independent of other cardiovascular risk factors. 15 The most recent literature investigating the dose-response relationship between stroke and Hcy levels was performed in 2002; however, this review did not differentiate between ischemic stroke and hemorrhagic stroke. 15 Therefore, we performed a systematic review and meta-analysis to assess the dose-response association between Hcy levels and the risk of ischemic stroke.

## Methods

The present systematic review and meta-analysis was conducted in accordance with the Preferred Reporting Items for Systematic Reviews and Meta-Analyses (PRISMA) guidelines. 16 The protocol was published in the Prospero database (ID: CRD42019130371).

### Literature Search

Searches in PubMed, Embase, Scopus, and Web of Science were performed on May 8, 2020. Where possible, filters were applied to remove nonhuman studies, and non-English language publications. No limits were set with regard to publication year. Free-text and the Medical Subject Headings (MeSH) terms or Emtree-preferred terms were used. Search combinations included terms related to the following search categories: Hcy, thromboembolism, biomarker, and adult human population. The complete search combinations used in PubMed is provided hereinafter. Similar search combinations were used for searches in the remaining three databases.

#### PubMed

Search ((((((((“Homocysteine”[Mesh] OR “Hyperhomocysteinemia”[Mesh] OR homocyst* OR hyperhomocyst*))) AND ((“Embolism and Thrombosis”[Mesh] OR “Myocardial Infarction”[Mesh] OR “Acute Coronary Syndrome”[Mesh] OR “Brain Ischemia”[Mesh] OR “Stroke”[Mesh] OR “Deep vein thrombosis” OR “pulmonary embolism” OR “lung embolism” OR thrombosis OR embolism OR thromboembolism OR stroke OR “acute stroke” OR “brain infarction” OR “cerebral infarction” OR “brain ischemia” OR “cerebral ischemia” OR “ischemic stroke” OR “intracranial embolism” OR “intracranial thrombosis” OR apoplexy OR “cerebrovascular accident” OR “cerebral stroke” OR “myocardial infarction” OR “myocardial infarct” OR “heart infarct” OR “heart infarction” OR “acute coronary syndrome” OR “acute myocardial infarction” OR “brain embolism” OR “cardiovascular stroke” OR “heart attack” OR “acute myocardial infarct” OR “acute heart infarction”))) AND ((“Biomarkers”[Mesh] OR “Blood”[Mesh] OR blood OR serum OR plasma OR biomarker OR “biological marker” OR “blood level” OR “blood levels”))) AND ((“Humans”[Mesh] OR adult[MeSH Terms] OR adults[All Fields] OR adult[All Fields] OR patients[All Fields] OR patient[All Fields] OR humans[All Fields] OR human[All Fields]))) AND ((Danish[Language] OR Norwegian[Language] OR English[Language] OR swedish[Language])))) NOT ((comment[Publication Type] OR congress[Publication Type] OR letter[Publication Type] OR “Case Reports”[Publication Type])).

**Fig. 1:**
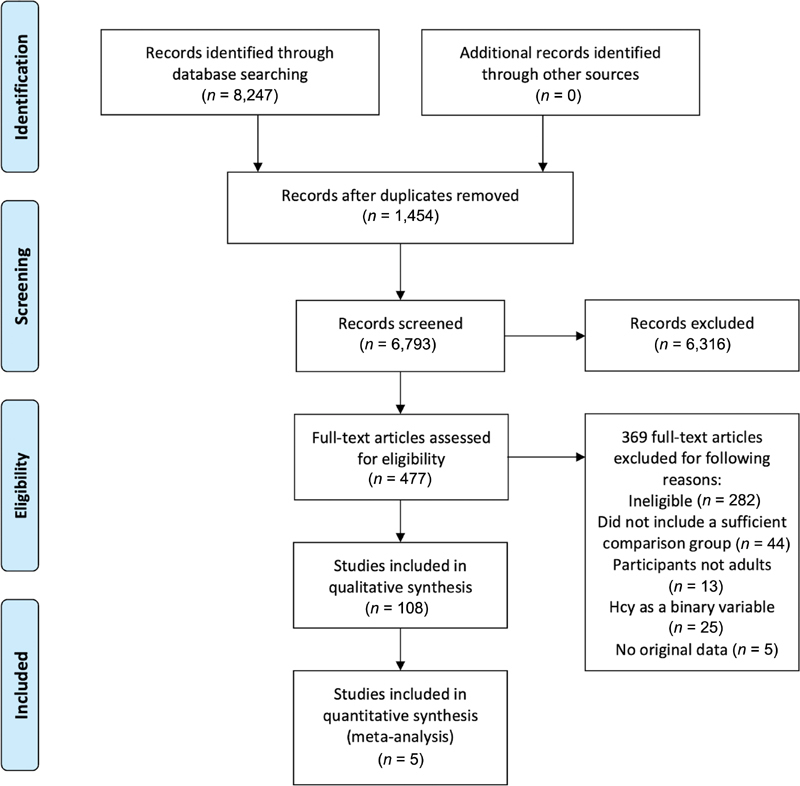
Preferred Reporting Items for Systematic Reviews and Meta-Analyses (PRIMSA) flow diagram. 16

Initially, all thromboembolic events were included as outcome, as seen in our search combinations, resulting in a large number of eligible articles ( Fig. 1 ). Therefore, our inclusion/exclusion criteria were revised after screening of abstracts to include only articles with ischemic stroke as outcome for this review. This choice was based on results found in the 2017 Cochrane review 14 which showed a potential association between Hcy and ischemic stroke in randomized trials of B-vitamin treatment.

Our inclusion criteria were studies investigating ischemic stroke events in patients with measured plasma Hcy providing original data on adult human populations. Both interventional and observational studies were included, including randomized trials, cohort, cross-sectional, and case-control studies. The accepted endpoint was acute ischemic stroke, including all subtypes. The exclusion criteria were as follows: nonoriginal literature, reviews, meta-analyses, guidelines, case studies, conference abstracts, and letters/editorials/comments without original data; missing information on Hcy concentration or studies including hyper-Hcy as a binary variable; endpoint of transient cerebral ischemia and arteriosclerotic lesions without sign of thrombosis, and silent brain infarction; and language other than English.

First, 100 abstracts were randomly selected and screened independently by the three authors for either exclusion or inclusion to full-text screening. Any disagreement was solved by consensus. Screening of the remaining abstracts was performed by MH. Similarly, 50 randomly selected articles proceeding to full text screening were read in full by all authors, and any disagreement was solved by consensus. Remaining papers were screened by MH for inclusion or exclusion, and in case of doubt, all three authors discussed the study in question.

### Data Extraction and Quality Assessment

Data extraction from the included articles was performed by M.H. and verified by A.M.H. and J.F.H.A. Study quality was assessed by all three authors using the Study Quality Assessment Tools for Observational Cohort and Cross-Sectional studies and for Case-Control studies, National Heart, Lung, and Blood Institute, National Institute of Health, the United States. 17 Each study was rated good, fair or poor according to the estimated risk of bias. Disagreement between authors was solved by consensus.

### Data Synthesis

We performed a meta-analysis of odds ratios (ORs) in which Hcy was included as a continuous linear variable. 18 19 20 21 22 23 Hcy increments was standardized to 5 µmol/L. A random effects model was used to obtain a summary OR as a measure of the relative risk. Forest plots and funnel plots were used to visualize the data.

## Results

In total, 108 original articles were included in the review. Of these, 22 articles rated good, 47 fair and 39 poor. Screening of abstracts and inclusion of articles are shown in Fig. 1 . Articles rated good were grouped according to study design of which 18 were case-control studies and 4 were observational cohort studies, as presented in Tables 1 , 2 , 3 . Only articles rated good are reported on and discussed in this article. Studies rated fair 24 25 26 27 28 29 30 31 32 33 34 35 36 37 38 39 40 41 42 43 44 45 46 47 48 49 50 51 52 53 54 55 56 57 58 59 60 61 62 63 64 65 66 67 68 and poor 69 70 71 72 73 74 75 76 77 78 79 80 81 82 83 84 85 86 87 88 89 90 91 92 93 94 95 96 97 98 99 100 101 102 103 104 are provided in Table 4 . For the dose-response analysis, we included articles rated fair to supplement results in articles rated good ( Table 5 ).

**Table 1:** Case-control studies investigating the association between ischemic stroke and homocysteine levels, *n* = 18

Study (year)	Study population Cases: *n* , mean age ± SD, %male Controls: *n* , mean age ± SD, %male	Hcy results (µmol/L) Timing of blood sampling a	Outcome and diagnostic criteria of ischemic stroke	Matched variables and adjusted covariates (Adj)
Campbell et al (2006) 110	Cases: *n* = 252, mean age = 67 ± 8 years 73% male Controls: *n* = 544, mean age = 66 ± 8 years 73% male	Cases, median Hcy: 16.3 µmol/L (IQR: 13.3–20.0) Controls, median Hcy: 16.3 µmol/L (IQR: 13.3–19.2) Timing: at baseline; prior to outcome Mean follow-up till outcome: 3.9 years	Outcome: ischemic stroke Diagnostic criteria: CT scan within 3 weeks, autopsy	Matched: age (within 5 years), sex, treatment allocated (perindopril-based/ placebo, mono/dual therapy), region, most recent qualifying event at randomization Adj: matched variables, systolic blood pressure, total cholesterol, current smoking, diabetes mellitus, peripheral arterial disease, antihypertensive medication other than β-blockers, calcium channel blockers, diuretics
Cui et al (2008) 19	Cases: n = 101, mean age = 68.7 years Male % = not stated Controls: *n* = 101, mean age = 67.7 Male % = not stated	Cases: 13.8 µmol/L Controls: 12.5 µmol/L Timing: prior to outcome (13–15 years)	Outcome: CVD, subclassified into ischemic stroke Diagnostic criteria: ICD9 codes 433–434, ICD10 code I63	Matched: sex, age, community, year of serum storage Adj: BMI, serum total and HDL-cholesterol, alcohol, smoking status, history of hypertension, diabetes
Eikelboom et al (2000) 23	Cases: *n* = 219, mean age = 66.1 ± 12.4 years 64% male Controls: *n* = 205 60% male, mean age = 67.0 ± 11.8 years	Cases: 12.4 µmol/L (11.7–13.2) Controls: 10.5 µmol/L (10.0–11.0) Timing: within 7 days of outcome	Outcome: ischemic stroke Diagnostic criteria: CT scan within 3 weeks, autopsy	Matched: none Adj: age, sex, creatinine, red cell folate, serum folate, pyridoxine cobalamin, MTHFR genotype, smoking, hypertension, diabetes mellitus, hypercholesterolemia, previous vascular events
Fallon et al (2003) 21	Study population: male smokers Cases: *n* = 212, mean age = 58.9 ± 5.2 years Controls: *n* = 212, mean age = 58.8 ± 5.3 years	Cases: 13.3 µmol/L (12.6–13.9) Controls: 12.6 µmol/L (12.0–13.2) Timing: prior to outcome	Outcome: ischemic stroke Diagnostic criteria: medical records, ICD9 codes 433–434 Register of causes of death	Matched: age (4-year range) Adj: all case/control pairs: systolic + diastolic blood pressures, total serum cholesterol, education, BMI, smoking: duration + cigarettes smoked daily + debut age, trial treatment group 120 case/control pairs: further adj. for serum folate, B6, alcohol
Haltmayer et al (2002) 20	Study population: male patients with symptomatic PAD Cases: *n* = 50, mean age = 69.8 years (25–75th percentile, 61.7–73.8) Controls: *n* = 400, mean age = 66.6 years (25–75th percentile, 57.6–73.1)	Cases median Hcy: 18.6 µmol/L (25–75th percentile, 13.7–23.1) Controls: 15.1 µmol/L (25–75th percentile, 12.4–18.5) Timing: after outcome (5 months–21 years)	Outcome: ischemic stroke Diagnostic criteria: medical reports + additional CT scans	Matched: none Adj: age, BMI, hypertension, diabetes mellitus, current smoking, carotid stenosis >50%, total cholesterol, HDL-cholesterol, serum triglycerides
Hultdin et al (2011) 111	Cases: *n* = 321 ischemic stroke *n* = 60 hemorrhagic stroke 55.8% male with ischemic stroke, mean age = 55.0 ± 8.1 years Controls: *n* = 788, 58.6% male, mean age = 55.0 ± 8.0 years	Cases: 12.8 µmol/L (±SD 5.6) Controls: 12.7 µmol/L (±SD 7.7) Timing: prior to outcome, average time to outcome >4 years	Outcome: first ever stroke, subclassified into ischemic and hemorrhagic stroke Diagnostic criteria: ICD-9 codes 430–438, CT, MRI scan, autopsy	Matched: age, sex Adj: BMI, hypertension
Iso et al (2004) 18	Cases: *n* = 90, 61% male, mean age = 65.9 years Controls: *n* = 294, 61% male, mean age = 66.0 years	Cases: 9.8 µmol/L (9.1–10.4) Controls: 9.0 µmol/L (8.7–9.4) Timing: at inclusion, years prior to outcome	Outcome: Stroke (subclassification: hemorrhagic, lacunar, large-artery occlusive, embolic) Diagnostic criteria: stroke identified with CT, ICD9 diagnosis codes 430–438, self-reporting	Matched: sex, age, community, year of serum stored, fasting status Adj: hypertension status, BMI, current alcohol intake, cigarette smoking status, serum total cholesterol levels, log-transformed triglyceride levels, quartiles of CRP, serum glucose category
Kaplan et al (2008) 112	Study population: Postmenopausal women Cases: *n* = 972, mean age = not stated Controls: *n* = 972, mean age = not stated	Cases, median Hcy: 8.5 µmol/L (IQR: 3.7) Controls, median Hcy: 8.2 µmol/L (25–75th percentile, 6.6–10.2) Timing: prior to outcome	Outcome: first ever ischemic stroke Diagnostic criteria: self-reporting, reports by family, medical records	Matched: age, race/ethnicity Adj: aspirin use, BMI, diabetes, systolic blood pressure, smoking, high cholesterol requiring medication, antihypertensive medication, fasting glucose, LDL, HDL
Khan et al (2008) 109	Study population: Afro-American population of the United Kingdom Total group: *n* = 457, 56% male, mean age = 65.4 ± 12.2 years Cases (ischemic stroke group): *n* = 408, Male % = not stated, mean age = not stated Controls (nonischemic stroke group): *n* = 179, 62.0% male, mean age = 65.4 ± 7.4 years	Cases: 14.3 µmol/L (±SD 8.8) Controls: 11.8 µmol/L (±SD 5.7) Timing: after outcome	Outcome: stroke, including subclassification Diagnostic criteria: CT or MRI scan. Subtyping of stroke using TOAST criteria	Matched: age and sex Adj: age, sex, hypertension, diabetes, hypercholesterolemia, smoking, B12, folate, eGFR
Li et al (2003) 115	Cases: *n* = 1,832 stroke patients; cerebral thrombosis: *n* = 807, lacunar infarction: *n* = 513, intracerebral hemorrhage: *n* = 503, 63.5% male, mean age = 60.3 ± 9.4 years Controls: *n* = 1,832, 57.4% male, mean age = 59.6 ± 8.8 years	Cases, median Hcy: cerebral thrombosis: 14.7 µmol/L (range: 207.8) Lacunar infarct: 14.8 µmol/L (range: 115.4) Controls, median Hcy: 12.8 µmol/L (range: 123.2) Timing: 6 weeks after outcome	Outcome: Stroke (subclassification: cerebral thrombosis, lacunar infarction, cerebral hemorrhage) Diagnostic criteria: CT/MRI scan, ICD 9 diagnosis codes 430–438	Matched: age (5-year range), community of residence Adj: age, sex, blood pressure, BMI, cigarette smoking, glucose, total cholesterol, triglycerides, glomerular filtration rate
Liang et al (2017) 114	Study population: 377 patients with essential hypertension Cases: *n* = 114, 60% male, mean age = 66.59 ± 11.15 years Hypertensive controls: *n* = 263, 64.6% male, mean age = 65.0 ± 11.29 years Normotensive controls: *n* = 109, 66.1% male, mean age = 66.13 ± 10.62 years	Cases: 19.11 µmol/L (±SD 9.70) Hypertensive controls: 13.24 µmol/L (±SD = 5.96) Normotensive controls: 12.78 µmol/L (±SD = 8.00) Timing: at admission for outcome	Outcome: ischemic stroke Diagnosis criteria: MRI scan within 24 hours	Matched: age and sex Adj: age, sex, systolic + diastolic blood pressure, cigarette smoking
Loffredo et al (2005) 22	Study population: 163 patients with nonvalvular atrial fibrillation Cases: *n* = 40, 40% male, mean age = 74.8 ± 8.8 years Controls: *n* = 123, 49.6% male, mean age = 69.2 ± 11.5 years	Cases: 18.1 µmol/L (±SD = 9.0) Controls: 15.4 µmol/L (±SD = 9.3) Timing: at inclusion, after outcome	Outcome: ischemic stroke, occurring >3 months prior to inclusion Diagnosis criteria: CT scan, medical records	Matched: none Adj: sex, hypertension, diabetes mellitus, dyslipidemia, smoking habits, prior coronary heart disease, left ventricular ejection fraction, left atrium diameter, oral anticoagulants, aspirin, predictors of tHcy, fibrinogen levels
Rueda-Clausen et al (2012) 113	Cases: *n* = 238, 55% male, mean age = 66.5 years (IQR: 58.1–75) Controls: *n* = 238, 55% male, mean age = 70.8 years (IQR: 61–77)	Cases, median Hcy: 10.01 µmol/L (IQR: 7.79–13.2) Controls, median Hcy: 8.48 µmol/L (IQR: 7.28–10.91) Timing: within 96 hours of onset of stroke symptoms	Outcome: ischemic stroke Diagnostic criteria: CT scan within 96 hours	Matched: age, sex, region of residence Adj: age, sex, pack year of smoking, plasma creatinine levels, waist to hip ratio, hypertension, diabetes mellitus, use of statins, socioeconomic status
Shimizu et al (2002) 106	Cases: *n* = 75, 52% male, mean age =75 years Controls: *n* = 248, 62% male, mean age = 71 years	Cases: 13.0 µmol/L Controls: 11.8 µmol/L Timing: 3 months–30 years (mean: 7.6 years) after outcome	Outcome: ischemic stroke (subclassification: lacunar, artherothrombotic, cardioembolic, undetermined) Diagnostic criteria: CT/MRI scan	Matched: age (2 years range), sex Adj: age, sex, hypertension, serum creatinine, total protein, folate, B12
Tan et al (2002) 108	Study population: young adults (20–50 years) Cases: *n* = 109, 71.6% male, mean age = 43.8 ± 5.87 years Controls: *n* = 88, 71.6% male, mean age = 43.1 ± 6.60 years	Cases: 13.7 µmol/L (12.7–14.9) Controls: 10.8 µmol/L (9.9–11.8) Timing: within 5 days of outcome	Outcome: first ever ischemic stroke Diagnostic criteria: CT/MRI scan within 1 week	Matched: age, sex Adj: age, sex, diabetes mellitus, hypertension, hyperlipidemia, B12
Tanne et al (2003) 105	Study population: 3,090 patients with preexisting chronic coronary artery disease Cases: *n* = 160, 95% male, mean age = 61.2 ± 6.3 years Controls: *n* = 160, 95% male, mean age =61.3 ± 6.4 years	Cases, median Hcy: 16.4 µmol/L (IQR: 12.7–14.3) Controls, median Hcy: 14.3 µmol/L (IQR: 12.0–17.8) Timing: prior to outcome	Outcome: Ischemic stroke, including subclassifications Diagnostic criteria: CT scan. Subtyping using TOAST criteria	Matched: age, sex, benzafibrate/placebo study medication (benzafibrate/placebo) Adj: Age, sex, BIP study medication, current smoking, diabetes mellitus, hypertension, previous myocardial infarction
Tascilar et al (2009) 107	Cases: large-vessel atherosclerotic stroke: *n* = 103, 68% male, mean age = 61.19 ± 14.20 years Cardioembolic stroke: *n* = 37, 45.9% male, mean age = 73.35 ± 10.72 years Controls: *n* = 37, 37.8% male, mean age = 53 ± 7.45 years	Cases: large-vessel atherosclerotic stroke: 13.94 µmol/L (±SD = 6.56) Cardioembolic stroke: 14.96 µmol/L (±SD = 5.94) Controls: 10.98 µmol/L (±SD = 2.91) Timing: within 24 hours of outcome	Outcome: large-vessel atherosclerotic stroke + cardioembolic stroke Diagnostic criteria: CT/MRI scan	Matched: none Adj: sex, smoking, hypertension, diabetes mellitus, hyperlipidemia
Verhoef et al (1994) 9	Patients: *n* = 109, mean age = 61.9 ± 9.1 years Controls: *n* = 427, mean age = 59.2 ± 8.9 years	Cases: 11.1 µmol/L (±SD = 4.0) Controls: 10.6 µmol/L (±SD 3.4) Timing: at inclusion, follow-up 5 years, outcome within these 5 years	Outcome: ischemic stroke Diagnostic criteria: medical reports, confirmed by CT scan, autopsy	Matched: age, smoking habits Adj: age, smoking habits, diabetes, hypertension, Queteet's index, aspirin assignment, total cholesterol-to-HDL cholesterol ratio, time since the last meal before the blood was drawn

Abbreviations: Adj, adjusted; BMI, body mass index; CRP, C-reactive protein; CT, computed tomography; CVD, cardiovascular disease; eGFR, estimated glomerular filtration rate; Hcy, homocysteine; HDL, high density lipoprotein; ICD, International Classification of Diseases; IQR, interquartile range; LDL, low density lipoprotein; MRI; magnetic resonance imaging; MTHFR, methylenetetrahydrofolate reductase; PAD, peripheral arterial disease; SD, standard deviation; TOAST, Trial of Org 10172 in Acute Stroke Treatment.

Note: Hcy levels is indicated as mean (95% confidence interval) unless other otherwise specified. Age is indicated as mean ± standard deviation unless other otherwise specified.

a Timing of blood sampling refers to time of blood sampling used to determinate Hcy concentration in subjects, indicating if blood sampling occurred prior to outcome or after outcome.

**Table 2:** Summary of results reported in studies analyzing dose-response relationship between ischemic stroke and homocysteine, *n* = 20

Study (year)	Outcome	Stratum 1 (µmol/L), effect measure (95% CI)	Stratum 2 (µmol/L), effect measure (95% CI)	Stratum 3 (µmol/L), effect measure (95% CI)	Stratum 4 (µmol/L), effect measure (95% CI)	Stratum 5 (µmol/L), effect measure (95% CI)	Increments, effect measure (95% CI)
Bostom et al (1999) 118	Nonhemorrhagic stroke	13–9.25 Ref.	9.26–11.43 RR = 1.22 (0.73–2.01)	11.44–14.23 1.31 (0.79–2.16)	14.24–219.84 1.79 (1.11–2.89)		
Bostom et al (1999) 118	Atherothrombotic brain infarction	13–9.25 Ref.	9.26–11.43 RR = 1.30 (0.68–2.49)	11.44–14.23 1.82 (0.99–3-36)	14.24–219.84 1.90 (1.02–3.51)		
Cui et al (2008) 19	Ischemic stroke	<10.5 Ref.	10.5–12.4 OR = 1.83 (0.54–6.28)	12.5–15.2 1.85 (0.57–5.98)	≥15.3 4.35 (1.12–16.9)		Per 5 µmol/L OR = 1.49 (1.01–2.18)
Eikelboom et al (2000) 23	Ischemic stroke						Per 5 µmol/L OR = 2.7 (1.4–5.1)
Fallon a et al (2003) 21	Ischemic stroke	3.1–10.5 Ref.	10.6–12.6 OR = 1.7 (0.9–3.1)	12.7–15.4 1.9 (1.1–3.2)	15.4–86.2 2.1 (1.1–3.9)		
Fallon b et al (2003) 21	Ischemic stroke	3.1–10.5 Ref.	10.6–12.6 OR = 1.2 (0.6–2.4)	12.7–15.4 1.9 (1.0–3.6)	15.4–86.2 2.0 (1.0–4.0)		Per 4.7 µmol/L OR = 1.4 (1.1–1.7)
Haltmayer et al (2002) 20	Ischemic stroke						Per 5.0 µmol/L OR = 1.37 (1.13–1.67)
Hultdin et al (2011) 111	Ischemic stroke	Ref. Ref.	Men:10.3 Women: 9.5 OR = 0.99 (0.63–1.54)	Men: 12.6 Women: 11.7 1.08 (0.70, 1.69)	Men: 15.3 Women: 14.3 0.86 (0.54–1.37)		
Iso et al (2004) 18	Ischemic stroke	4.1–7.0 Ref.	7.0–8.7 OR = 1.36 (0.60–3.09)	8.7–11.0 1.45 (0.60–3.49)	11.0–47.3 3.89 (1.60–9.46)		Per 5 µmol/L OR = 1.52 (1.07–2.14)
Kaplan et al (2008) 112	First ever ischemic stroke	<6.6 Ref.	– OR = 1.15 (0.86–1.52)	– 1.23 (0.93–1.64)	>10.4 1.26 (0.95–1.68)		
Khan et al (2008) 109	Ischemic stroke						Per 1 µmol/L increase in log Hcy: OR = 4.02 (2.13–7.57)
Liang et al (2017) 114	Ischemic stroke						Per SD increase in log Hcy: OR = 1.62 (1.17–2.25)
Loffredo et al (2005) 22	Ischemic stroke	4.6–7.5 Ref.	9.7–14.1 OR = 0.75 (0.31–1.82)	14.3–18.6 1.30 (0.55–3.07)	18.7–67.1 2.73 (1.23–6.08)		Per 1 µmol/L OR = 1.056 (1.00–1.12)
Petri et al (1996) 117	Ischemic stroke						Per 1 unit in log Hcy: OR = 2.44 (1.04–5.75)
Rueda-Clausen et al (2012) 113	Ischemic stroke	≤12.69 Ref.	>12.69 OR = 8.97 (4.07–19.75)				
Shi et al (2018) 116	Ischemic stroke	≤9.65 Ref.	9.65 ≤ 11.9 HR = 0.77 (0.42–1.40)	11.9 ≤ 15.5 1.52 (0.89–2.62)	>15.5 1.76 (1.11–3.08)		
Shimizu et al (2002) 106	Ischemic stroke	<10.4 Ref.	10.4–13.6 OR = 2.0 (0.9–4.4)	≥13.6 4.0 (1.8–8.9)			
Tan et al (2002) 108	First ever ischemic stroke	<9.6 Ref.	9.6–12.0 OR = 0.94	12.1–14.95 3.2	>14.95 4.3 (1.5–12.6)		Per 1 µmol/L increase in log Hcy: OR = 5.17 (1.96–13.63)
Tanne et al (2003) 105	Ischemic stroke	<11.4 Ref.	11.4–13.2 OR = 1.48 (0.44–5.46)	13.3–17.4 2.11 (0.58–8.75),	>17.4 4.62 (1.32–18.86)		Per 1 µmol/L increase in ln Hcy: OR = 3.41 (1.08–12.30)
Tascilar et al (2009) 107	Large-vessel atherosclerotic stroke	4.00–9.20 Ref.	9.21–12.40 OR = 0.813 (0.286–2.310)	12.70–15.80 1.285 (0.406–4.067)	15.90–42.80 2.449 (0.660–9.095)		
Tascilar et al (2009) 107	Cardio-embolic stroke	4.00–9.20 Ref.	9.21–12.40 OR = 0.805 (0.191–3.392)	12.70–15.80 1.929 (0.471–7.902)	15.90–42.80 5.745 (1.271–25.959)		
Verhoef et al (1994) 9	Ischemic stroke	≤12.7 Ref.	>12.7 OR = 1.2 (0.7 - 2.0)				
Zee et al (2007) 119	Ischemic stroke	<8.47 Ref.	8.48–9.97 HR = 1.02 (0.63–1.64)	9.98–11.55 1.24 (0.79–1.96)	11.56–14.04 1.01 (0.63–1.62)	>14.05 1.27 (0.80–2.00)	

Abbreviations: CI, confidence interval; Hcy, homocysteine; HR, hazard ratio; OR, odds ratio; Ref., reference value; RR, risk ratio; SD, standard deviation.

Notes: numbers in parentheses after RR/OR/HR are 95% CI unless otherwise specified.

a Results from model A in Fallon et al: 201 matched case-control pairs.

b Results from model C in Fallon et al: 120 cases, 310 controls, unmatched.

**Table 3:** Cohort studies investigating the association between ischemic stroke and homocysteine levels, *n* = 4

Study (year)	Study population: *n* , % male, mean age Follow-up time	Hcy-results (µmol/L) Timing of blood sampling a	Outcome and diagnostic criteria of ischemic stroke	Adjusted covariates
Bostom et al (1999) 118	Study population: elderly patients, *n* = 1947, 40.5%male, mean age = 70 ± 7 years Follow-up time: mean = 9.9 years	Mean Hcy: 12.65 ± 7.19 µmol/L Timing: at study inclusion, prior to outcome	Outcome: total stroke, no-hemorrhagic stroke, atherothrombotic brain infarctions Diagnostic criteria: CT scan	Age, sex, diabetes, cigarette smoking, systolic blood pressure, prior coronary heart disease, prior atrial fibrillation
Shi et al (2018) 116	Study population: acute stroke patients Ischemic stroke: *n* = 2,587, 70.0% male, mean age = 60.7 ± 10.5 years Follow-up time: median 18 months	Hcy: within 3 days of ischemic stroke: 14.4 ± 10.3 µmol/L 3 months after ischemic stroke: 14.3 ± 10.0 µmol/L Timing: within 24 hours of outcome, and again three months after	Outcome: recurrence of ischemic stroke, including subclassification Diagnostic criteria: CT scan. Subtyping using TOAST criteria	Age, sex, smoking status, low-density lipoprotein cholesterol level, CRP level, apolipoprotein B/Apolipoprotein AI ratio, presence of hypertension, type-2 diabetes mellitus, coronary artery disease, obesity
Petri et al (1996) 117	Study population: systemic lupus erythematosus patients Cases: *n* = 29, 14%male, mean age = 38.6 ± 15.2 years Controls: *n* = 308, 7.1% male, mean age = 34.5 ± 11.3 years Follow-up time: 1,619 person-years (mean 4.8 ± 1.7 years)	Cases: 10.26 ± 1.91 µmol/L Controls: 7.41 ± 1.88 µmol/L Timing: at inclusion, prior to outcome	Outcome: stroke, arterial or venous thrombotic events Diagnostic criteria of ischemic stroke: CT/MRI scan	Age, sex, race, obesity, hypercholesterolemia, hypertension, diabetes, renal insufficiency, lupus anticoagulant
Zee et al (2007) 119	Study population: healthy white women: *n* = 24,968 Mean age stratified for MTHFR genotype (CC, CT, TT): CC = 54.7 ± 7.1 years; CT = 54.7 ± 7.1 years; TT = 54.7 ± 7.2 years Follow-up time: mean follow-up of 9.9 ± 1.3 years, 246, 852 person-years	Hcy: stratified for MTHFR genotype (CC, CT, TT): 11.1 ± 4.3 µmol/L, 11.4 ± 4.9 µmol/L, 12.5 ± 6.1 µmol/L Timing: at inclusion, prior to outcome	Outcome: ischemic stroke Diagnostic criteria: medical records, the National Death Index, autopsy reports, death certificates, reports from family	Age, smoking status, systolic blood pressure, total cholesterol, HDL cholesterol, diabetes mellitus, hormone use

Abbreviations: CRP, C-reactive protein; CT, computed tomography; CVD, cardiovascular disease; Hcy, homocysteine; HDL, high density lipoprotein; MRI; magnetic resonance imaging; MTHFR, methylenetetrahydrofolate reductase; TOAST, Trial of Org 10172 in Acute Stroke Treatment.

Note: Hcy levels are indicated as mean ± standard deviation unless other otherwise specified. Age is indicated as mean ± standard deviation unless other otherwise specified.

a Timing of blood sampling, refers to time of blood sampling used to determinate Hcy concentration in subjects, indicating if blood sampling occurred prior to outcome or after outcome.

**Table 4:** Rating of individual studies that did not include a dose-response analysis rated fair or poor

Study (year) Rated fair	Study (year) Rated poor
Case-control studies	Case-control studies
Bosco et al (2006) 25	Al-Allawi et al (2009) 71
Kelly et al (2004) 31	Alkali et al (2006) 131
Kim et al (2011) 32	Araki et al (1989) 72
Kim et al (2011) 33	Fekih-Mrissa et al (2013) 80
Kristensen et al (1999) 34	Ma et al (2017) 42
Lee et al (2008) 36	Sun et al (2005) 95
Li et al (2018) 38	Tas et al (2005) 96
Lu et al (2018) 40	Yi et al (2013) 101
Luo et al (2017) 41	Yingdong et al (2002) 102
Ma et al (2011) 43	Cross-sectional studies
Mao and Han (2018) 44	Adunsky et al (2000) 69
Meiklejohn et al (2001) 45	Ben-Salem et al (2010) 73
Modi et al (2005) 47	Brattström et al (1992) 74
Moe et al (2008) 48	Cao et al (2019) 128
Mojiminiyi et al (2008) 49	Celikbilek et al (2014) 75
Pezzini et al (2002) 52	Cingozbay et al (2002) 76
Rahman et al (2013) 54	Coull et al (1990) 77
Tantirittisak et al (2007) 55	El Kossi and Zakhary (2000) 78
Vayá et al (2011) 57	Fatima et al (2012) 79
Yang et al (2004) 62	Karakurum Goksel et al (2007) 81
Yang et al (2016) 63	Han et al (2002) 82
Zhang et al (2014) 66	Karabulut et al (2017) 83
Zhang et al (2019) 67	Kokocińska et al (2005) 84
*Cohort studies*	Li et al (2004) 85
Press et al (1999) 53	Liu et al (2005) 86
Cross-sectional studies	Moghaddasi et al (2010) 87
Dai et al (2020) 129	Mykhalojko and Mykhalojko (2017) 88
Haapaniemi et al (2007) 27	Narang et al (2009) 89
Kara et al (2009) 29	Omrani et al (2011) 90
Kucukarabaci et al (2008) 35	Peng et al (2001) 91
Lehmann et al (2015) 37	Sawuła et al 2009 92
Lindgren et al (1995) 39	Sun et al (2009) 94
Mejia et al (2011) 46	Sönmezler et al (2013) 93
Ustundag et al (2010) 56	Unal et al (2013) 97
Wei et al (2019) 130	Urbańska et al (2006) 98
Xia et al (2014) 60	Wei et al (2018) 99
Yao et al (2017) 64	Wu et al (2017) 100
Zhu et al (2013) 68	Zhang et al (2014) 103
	Zhou et al (2005) 104

**Table 5:** Summary of results reported in studies rated fair analyzing dose-response relationship between ischemic stroke and homocysteine

Study (year)	Outcome	Stratum 1 (µmol/L), effect measure (95% CI)	Stratum 2 (µmol/L), effect measure (95% CI)	Stratum 3 (µmol/L), effect measure (95% CI)	Stratum 4 (µmol/L), effect measure (95% CI)	Increments, effect measure (95% CI)
Case-control studies						
Atanassova et al (2007) 24	Ischemic stroke	–	–	–	–	Per 1 µmol/L OR = 1.22 (1.03–1.44)
Delport et al (1997) 26	Ischemic stroke	>10.53 Ref.	<10.53 OR = 3.7 (0.8–16.7)	–	–	–
Hassan et al (2004) 28	Lacunar infarction	>10.3 Ref.	10.3–13.0 OR = 1.42 (0.70–2.89)	13.1–15.9 2.02 (1.37–2.99)	>15.9 2.06 (1.53–2.78)	–
Ma (2017) a 42	Ischemic stroke	4.29–10.7 Additive model: OR = 0.43 (0.25–0.75) Ressicive model: OR = 0.09 (0.01–0.79) Dominant model: OR = 0.43 (0.23–0.82)	10.74–13.71 1.15 (0.69–1.92) 2.61 (0.74–9.18) 0.97 (0.51–1.84)	13.73–53.99 0.69 (0.42–1.14) 0.84 (0.21–3.29) 0.62 (0.35–1.11)	–	–
Parnetti et al (2004) 50	Ischemic stroke	–	–	–	–	Per 1 µmol/L OR = 1.425 (1.300–1.562)
Perini et al (2005) 51	Ischemic stroke	0–10 Ref.	10.1–13.2 OR = 2.1 ( *p* < 0.001)	13.3–18.6 2.8 ( *p* < 0.001)	>18.6 6.74 (3.78–12.02)	–
Perini et al (2005) 51	Small artery stroke	0–10 Ref.	10.1–13.2 OR = 3.9 (1.6–8.2)	13.3–18.6 5.9 (2.6–14.4)	>18.6 16.4 (6.9–44.3)	–
Perini et al (2005) 51	Large artery stroke	0–10 Ref.	10.1–13.2 OR = 1.5 (0.8–2.6)	13.3–18.6 2.7 (1.4–4.7)	>18.6 4.9 (2.4–9.8)	–
Perini et al (2005) 51	Cardioembolic stroke	0–10 Ref.	10.1–13.2 OR = 1.6 (0.7–3.4)	13.3–18.6 3.0 (1.3–6.4)	>18.6 7.1 (3.6–22.1)	–
Wang et al (2015) 58	Ischemic stroke	<15 Ref.	≥15 OR = 0.99 (0.64–1.51)	–	–	Per 5 µmol/L OR = 1.15 (1.01–1.28)
Yadav et al (2017) 61	Ischemic stroke	<12 Ref.	≥12 OR = 0.37 (0.16–0.83)	–	–	–
Yoo et al (1998) 65	Ischemic stroke	<15.5 Ref.	≥15.5 OR = 1.70 (1.48–1.95)	–	–	–
Cross-sectional studies						
Kario et al (2001) 30	Ischemic stroke	–	–	–	–	Per 1 SD increase OR = 2.16 (1.30–3.59)
Wang et al (2014) 59	Ischemic stroke	<15 Ref.	15–30 OR = 0.80 (0.59–1.074)	>30 OR = 0.91 (0.49–1.67)	–	Per 5 µmol/L OR = 0.99 (0.92–1.06)

Abbreviations: CI, confidence interval; OR, odds ratio; SD, standard deviation.

Note: numbers in parentheses after OR are 95% confidence intervals unless other otherwise specified.

a Ma et al divided participants in regard to their *EPHX2* G860A genotype into grouping of the Additive, Ressicive and Dominant genotype model.

### Case-Control Studies

Of the 18 case-control articles rated as good, seven studies subclassified ischemic stroke 18 105 106 107 108 109 ; 1 reported CVD with subanalyses for ischemic stroke 19 and 10 studies performed no subclassification of ischemic stroke. 9 20 21 22 23 110 111 112 113 114

The timing of blood sampling for measurement of Hcy levels varied among studies. Eight studies performed blood sampling prior to outcome 9 18 19 21 105 110 111 112 ; 10 studies performed blood sampling after outcome, 20 22 23 106 107 108 109 113 114 115 of which four studies collected blood within 7 days of outcome, 23 108 113 114 and 2 studies within 24 hours of outcome. 107 116

#### Studies with Effect Measures Based on Homocysteine Strata

Eleven studies stratified Hcy levels into several strata, estimating the risk of ischemic stroke in the lowest stratum compared with the higher strata ( Table 2 ). 18 19 21 22 23 105 106 107 108 111 112 Nine out of 11 studies found an increased risk of ischemic stroke when comparing patients in the highest versus lowest Hcy level strata. 18 19 21 22 23 105 106 107 108 Two studies found no association between risk of ischemic stroke and Hcy level 111 112 and one study reported an association for cardioembolic stroke, but not with large-vessel atherosclerotic stroke. 107 Two studies estimated effect measures using a dichotomous Hcy; one study found an association, 113 whereas one did not. 9

#### Studies with Effect Measures Based Homocysteine Increments

Ten studies included Hcy as a continuous variable and presented effect measures based on various increments of Hcy ( Table 2 ). 18 19 20 21 22 23 105 108 109 114 All studies found an association between increasing Hcy levels and odds of ischemic stroke, despite variations in the Hcy level increments that were employed.

Six studies included analysis of ischemic stroke subclasses. 23 105 106 107 108 109 Among these, four studies showed an association between small-vessel disease and/or large-vessel disease, 23 106 108 109 and three studies demonstrated an association with cardioembolic stroke. 105 107 109

Two studies did not include effect measures illustrating the dose-response association between ischemic stroke and Hcy levels. 110 115

Overall, in studies comparing Hcy strata, effect measures were clearly elevated when Hcy level reached 15 µmol/L and above ( Table 2 ).

### Cohort Studies

Four cohort studies were rated as good, ( Table 3 ). 116 117 118 119 Mean follow-up time ranged from 18 months to 9.9 years. Two studies included patients with CVD, with subanalyses for ischemic stroke. 117 119 Zee et al did not find an association when comparing quintiles of Hcy levels in the population, 119 whereas Petri et al found increased risk of ischemic stroke with increasing Hcy levels. 117 Bostom et al included elderly patients with stroke and found an association for both nonhemorrhagic stroke and atherothrombotic brain infarction, when comparing the highest quartile of Hcy to the lowest quartile. 118 Shi et al investigated recurrence of ischemic stroke as outcome, with enrolment at admission for first ever stroke. Blood sampling was performed at 3 days and 3 months after enrollment. An association between the risk of recurrent ischemic stroke (within 12–36 months) and Hcy levels was found, when comparing the highest and lowest Hcy quartiles in blood samples performed 3 months after the enrollment. 116

### Meta-analysis of Dose-Response Association

**Fig. 2:**
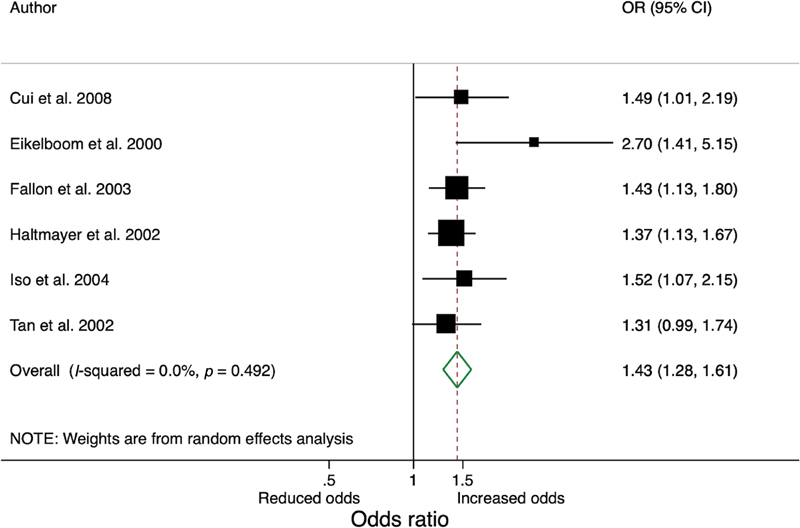
Forest plot of risk of ischemic stroke per 5 µmol/L increase in plasma homocysteine. CI, confidence interval; OR, odds ratio.

**Fig. 3:**
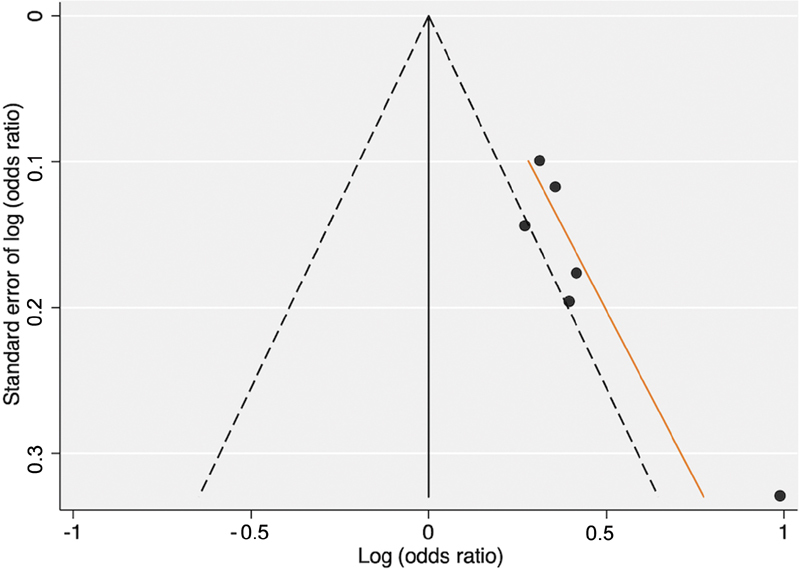
Funnel plot of studies included in meta-analysis investigating the risk of ischemic stroke per 5 µmol/L increase in homocysteine.

Eleven studies included Hcy as a continuous variable of which six were included in the meta-analysis. 18 19 20 21 22 23 We normalized ORs to increments of 5 µmol/L in Hcy. 21 22 The remaining five studies performed log transformation of Hcy levels prior to statistical analysis and were therefore not included in the meta-analysis ( Table 2 ). 106 108 109 114 117 The studies included in the meta-analysis reported similar results and included similar numbers of patients. All adjusted for age, sex, main CVD risk factors (diabetes, hypertension, hypercholesterolemia, smoking, and body mass index [BMI]), with an exception of Eikelboom et al that did not adjust for BMI. Eikelboom et al was the only study that adjusted for renal insufficiency ( Tables 1 and 3 ). 23 We performed a random effect analysis, resulting in an OR of 1.43 (95% confidence interval [CI]: 1.28–1.61; *I* ^2^ = 0.0%, *p* = 0.492; Fig. 2 ). A funnel plot for the meta-analysis is provided in Fig. 3 , as the resulting funnel plot was severely asymmetric.

Results reported in studies rated fair and further supported the observations reported in studies rated good ( Table 5 ).

## Discussion

The present study indicates a dose-response association between Hcy levels and the risk of ischemic stroke. It was apparent that risk estimates reported in studies were notably higher when reaching Hcy levels above 15 µmol/L, indicating a possible nonlinear association between Hcy and ischemic stroke. Both studies rated good and fair supported this observation.

Studies have shown that Hcy levels increase in patients within 1-week poststroke. 27 39 45 This could explain some of the differences observed between cases and controls in the case-control studies where blood sampling was performed in cases during hospitalization for stroke. 27 74 Moreover, Hcy levels have been shown to increase in critically ill patients. 120 In this review, no difference in means was observed when comparing Hcy levels in blood samples obtained during the acute phases 23 107 108 114 116 and convalescence phases of ischemic stroke 20 22 106 109 ; but based on the aforementioned previous studies, timing of blood sampling should be considered when evaluating Hcy as an exposure.

Five studies showed an association between small- and large-vessel diseases when subclassifying stroke using the Trial of Org 10172 in Acute Stroke Treatment (TOAST) criteria ( Tables 1 and 3 ). 23 106 108 109 116 This finding indicates that the effect of Hcy could depend on the underlying etiologically of ischemic stroke. Large randomized controlled trails investigating the effect of Hcy-lowering B-vitamin treatment have not demonstrated an effect on vascular outcomes or stroke. 11 12 121 Notably, reevaluation of data suggests that the effect of Hcy-lowering treatment could vary between outcomes, with a more beneficial effect on stroke than other CVD outcomes. 14 122 Subclassifying stroke further could help clarifying which etiologies of stroke are affected by Hcy, and which patients could potentially benefit from Hcy-lowering treatment. As such, the clinical relevance of assessing Hcy in stroke patients or screening for hyperhomocysteinemia to prevent stroke remains undetermined. Taken together with the conflicting results on the effect of Hcy-lowering vitamin treatment, this may also explain why measuring plasma Hcy is not recommended in most clinical guidelines on ischemic stroke. 11 12 13 14

The most recent review of the literature found a 59% increased risk of stroke when Hcy increased 5 µmol/L. 15 We report a similar increased risk of 43% (95% CI: 1.28–1.61) when standardizing reported ORs and using the same Hcy increments of 5 µmol/L. 18 19 20 21 22 23

The studies included in the present review assumed a linear association between Hcy levels and ischemic stroke; however, without describing this further or commenting on the hidden assumption of a linear association. As we observed a clear elevation in risk when surpassing 15 µmol/L, our results question this assumption of a linear association. Additionally, several studies performed a logarithmic transformation of Hcy levels prior to statistical analysis, indicating that they initially observed a nonlinear association with ischemic stroke risk, but without exploring this further.

To assess publication bias, we performed a funnel plot of our meta-analysis ( Fig. 3 ). Generally, at least 10 studies with varying sample sizes should be included for the test to have the power to distinguish chance from true asymmetry. Even though only six studies were included in the meta-analysis, we included the funnel plot as it was severely asymmetric. This could indicate publication bias, but it may also result from an overestimation of the effect of Hcy because of small study populations. 123

Several large randomized controlled trials of Hcy-lowering treatment report mainly no effect on risk of CVD. 12 13 14 124 These studies were excluded in the inclusion process of this review, as Hcy levels were not reported in the studies.

## Strengths and Limitations

One of the strengths of the present systematic review was the strict requirement of outcome definition required for inclusion. Numerous studies investigating the relationship between Hcy and stroke do not differentiate between hemorrhagic or ischemic stroke in their outcome variable which could lead to a reduction of estimates toward the null. 10 125 126 Second, this review only included multivariable adjusted risk estimates.

Some limitations have to be considered as well. First, statistical analyses were not standardized across studies and a meta-analysis of the dose-response relationship was only based on six studies. Second, the strategies for choosing control groups varied between hospital- and community-based controls; this could lead some studies to include a healthier control group compared with others. Third, we were not able to take into account differences in laboratory methods for measuring Hcy levels, and in turn, differences in reference intervals between studies. Forth, Hcy levels are influenced by a vast array of environmental and genetic factors, but most studies only adjusted effect measures for the main known cardiovascular risk factors, age, sex, hypertension, diabetes, cholesterol, smoking status, and BMI. However, six studies adjusted for renal function 23 106 109 113 115 117 and additional nutritional factors, such as folate and vitamin B12 levels, were adjusted in six studies. 21 22 23 106 108 109 We were not able to further assess the possible differential impact of the etiology of elevated Hcy levels and the association with ischemic stroke. Furthermore, lipid-lowering medication, such as fibrates, commonly prescribed for patients in risk of CVD, might influence the Hcy levels. 127 Any potential influence of lipid-lowering drugs on the association between Hcy and ischemic stroke was not assessed.

## Conclusion

The present review and meta-analysis indicate that a nonlinear association could exist between Hcy levels and the risk of ischemic stroke. This implies that the risk of ischemic stroke increases when Hcy exceeds a certain level. Identifying this cut-off point would be of strong clinical interest, as it could help distinguish which patients could benefit of Hcy-lowering treatment.
